# Codon usage patterns of *LT-Ag* genes in polyomaviruses from different host species

**DOI:** 10.1186/s12985-019-1245-2

**Published:** 2019-11-14

**Authors:** Myeongji Cho, Hayeon Kim, Hyeon S. Son

**Affiliations:** 1 Laboratory of Computational Biology & Bioinformatics, Institute of Public Health and Environment, Graduate School of Public Health, Seoul National University, 1 Gwanak-ro, Gwanak-gu, Seoul, 08826 South Korea; 2 Department of Biomedical Laboratory Science, Kyungdong University, 815 Gyeonhwon-ro, Munmak, Wonju, Gangwondo 24695 South Korea; 3 SNU Bioinformatics Institute, Interdisciplinary Graduate Program in Bioinformatics, College of Natural Science, Seoul National University, 1 Gwanak-ro, Gwanak-gu, Seoul, 08826 South Korea

**Keywords:** Polyomavirus, LT-Ag, Functional domains, Sequence motif, Codon usage pattern, RSCU

## Abstract

**Background:**

Polyomaviruses (PyVs) have a wide range of hosts, from humans to fish, and their effects on hosts vary. The differences in the infection characteristics of PyV with respect to the host are assumed to be influenced by the biochemical function of the LT-Ag protein, which is related to the cytopathic effect and tumorigenesis mechanism via interaction with the host protein.

**Methods:**

We carried out a comparative analysis of codon usage patterns of large T-antigens (LT-Ags) of PyVs isolated from various host species and their functional domains and sequence motifs. Parity rule 2 (PR2) and neutrality analysis were applied to evaluate the effects of mutation and selection pressure on codon usage bias. To investigate evolutionary relationships among PyVs, we carried out a phylogenetic analysis, and a correspondence analysis of relative synonymous codon usage (RSCU) values was performed.

**Results:**

Nucleotide composition analysis using *LT-Ag* gene sequences showed that the GC and GC3 values of avian PyVs were higher than those of mammalian PyVs. The effective number of codon (ENC) analysis showed host-specific ENC distribution characteristics in both the *LT-Ag* gene and the coding sequences of its domain regions. In the avian and fish PyVs, the codon diversity was significant, whereas the mammalian PyVs tended to exhibit conservative and host-specific evolution of codon usage bias. The results of our PR2 and neutrality analysis revealed mutation bias or highly variable GC contents by showing a narrow GC12 distribution and wide GC3 distribution in all sequences. Furthermore, the calculated RSCU values revealed differences in the codon usage preference of the *LT-AG* gene according to the host group. A similar tendency was observed in the two functional domains used in the analysis.

**Conclusions:**

Our study showed that specific domains or sequence motifs of various PyV LT-Ags have evolved so that each virus protein interacts with host cell targets. They have also adapted to thrive in specific host species and cell types. Functional domains of LT-Ag, which are known to interact with host proteins involved in cell proliferation and gene expression regulation, may provide important information, as they are significantly related to the host specificity of PyVs.

## Background

Polyomaviruses (PyVs) are non-enveloped double-stranded DNA viruses; a total of 86 PyV species have been classified by the International Committee on Taxonomy of Viruses. The classified member species belong to four genera, i.e., Alphapolyomavirus (36), Betapolyomavirus (32), Deltapolyomavirus (4), and Gammapolyomavirus (9), within the family Polyomaviridae (unassigned), while a genus of five species has not yet been classified. Their hosts are diverse, including humans, non-human primates (chimpanzees, gorillas, orangutans, and monkeys), non-primate mammals (bats, mice, racoon, badgers, cows, horses, elephants, alpacas, sea lions, seals, and dolphins), avian species (penguins, geese, and birds), and fish (sharks, perch, and cod) (https://talk.ictvonline.org/ictv-reports/ictv_online_report/dsdna-viruses/w/polyomaviridae).

The first PyV discovered was mouse PyV (MPyV), which was isolated from a murine tumor [1, 2] in the mid-1950s. Since then, simian virus 40 (SV40) was discovered in the renal cells of rhesus monkeys in the 1960s [3]. As mostly animal viruses were studied, the viruses seemed to be irrelevant to human diseases. However, two human PyVs, BKPyV and JCPyV, were found [4, 5], and in 2008, MCPyV was identified in human Merkel cell carcinoma tissue [6]. Thus, the various animal and human PyVs reported so far have drawn renewed attention. Most mammalian PyVs do not directly cause severe acute disease in infected hosts. However, an inconspicuous primary infection can persist for a lifetime, and when the host is in an immunosuppressed or immunocompromised state, such infection can lead to multiple diseases, such as progressive multifocal leukoencephalopathy and hemorrhagic cystitis, due to virus reactivation [7, 8]. PyV has a strong species-specific tendency, similar to papillomavirus [9, 10], and is thought to have co-evolved with amniotes. Various studies have been carried out to determine the infection characteristics of PyV. Therefore, it is necessary to understand their evolutionary history and their interaction with their hosts, as well as to interpret their genetic information.

Early and late gene RNAs of PyVs encode two and three proteins, respectively. The early gene is translated into 2 T-antigens (large T-antigen (LT-Ag) and small T-antigen), and the late gene is translated into three capsid proteins (VP1, VP2, and VP3) [11]. Among these, LT-Ag is directly related to tumorigenesis. Notably, the LT-Ag protein is known to bind to the p53 and Rb proteins, which are products of two typical tumor suppressor genes [12]. It has also been found to be a major factor determining the biochemical function of SV40 and MCPyV, which cause tumors in rodents and humans [13, 14]. The LT-Ag of PyV has functionally conserved domains, such as the DnaJ domain, LXCXE motif, NLS domain, Helicase domain, and p53 binding domain, that are present in most virus species [13]. Among these, the DnaJ domain, LXCXE motif, and p53 binding domain bind to proteins belonging to the cellular Hsc70 and Rb family and p53 cellular suppressor proteins, respectively, affecting replication and proliferation of the viral genome through DNA binding, ATP-dependent helicase, and ATPase activity. Specifically, when the early gene *LT-Ag* is continuously expressed, although PyV cannot to replicate its genome in nonpermissive hosts, cell transformation is induced, resulting in tumorigenesis. Each domain is considered to play an important role in this carcinogenesis.

PyVs vary in terms of toxicity to hosts, so their effects on hosts differ (Table 1). Variations in the infection characteristics of these viruses (whether they induce tumors due to binding to host proteins) among various hosts indicate the importance of the biochemical function of the LT-Ag protein in relation to host range and tumorigenesis. Therefore, in this study, we performed codon usage pattern, sequence similarity, and phylogenetic analyses using the genetic information of *LT-Ag* gene coding sequences (CDS) and major domains, to compare genetic characteristics. Based on the results of these analyses, we investigated the differences in the codon usage patterns depending on the taxon and PyV host and identified the relationships between phylogeny and sequence similarity among viruses. The genetic and evolutionary differences among the viruses identified by the comparative analysis offer a basis for explaining variations in their host range and toxicity. Based on these results, it is possible to infer the causes of the functional differences in LT-Ag among various PyVs.

**Table 1:** Proven and possible diseases associated with PyVs

Host	Virus name	Species	Abbr.	Clinical correlate	Ref.
Human	Merkel cell polyomavirus	Human polyomavirus 5	MCPyV	Merkel cell cancer	[6]
Human	Trichodysplasia spinulosa-associated polyomavirus	Human polyomavirus 8	TSPyV	Trichodysplasia spinulosa	[15]
Human	BK polyomavirus	Human polyomavirus 1	BKPyV	Polyomavirus-associated nephropathy; haemorrhagic cystitis	[4]
Human	JC polyomavirus	Human polyomavirus 2	JCPyV	Progressive multifocal leukoencephalopathy (PML)	[5]
Human	Human polyomavirus 6	Human polyomavirus 6	HPyV6	HPyV6 associated pruritic and dyskeratotic dermatosis (H6PD)	[16]
Human	Human polyomavirus 7	Human polyomavirus 7	HPyV7	HPyV7-related epithelial hyperplasia	[16]
Monkey	Simian virus 40	*Macaca mulatta* polyomavirus 1	SV40	PML-like disease in Immunocompromised animals	[3]
Hamster	hamster polyomavirus	*Mesocricetus auratus* polyomavirus 1	HaPyV	Skin tumors	[17]
Mouse	mouse pneumotropic virus	*Mus musculus* polyomavirus 2	MPtV	Respiratory disease in suckling mice	[18]
Bird	budgerigar fledgling disease virus	Aves polyomavirus 1	BFDV	Budgerigar fledgling disease; polyomavirus disease	[19–21]
Finch	Finch polyomavirus	*Pyrrhula pyrrhula* polyomavirus 1	FPyV	Polyomavirus disease	[22]
Goose	Goose hemorrhagic polyomavirus	*Anser anser* polyomavirus 1	GHPV	Hemorrhagic nephritis and enteritis	[23]

References are specified for first description

## Methods

### Data acquisition

The virus name, abbreviation, and classification information of 86 species belonging to the family Polyomaviridae were checked (https://talk.ictvonline.org/ictv-reports/ictv_online_report/dsdna-viruses/w/polyomaviridae), and the reference sequences were downloaded from the National Center for Biotechnology Information GenBank® (https://www.ncbi.nlm.nih.gov) (Table 2). The CDS regions of the *LT-Ag* genes to be analyzed were extracted and classified into the following five groups, according to the host of each virus species: non-primate mammals (Group M); non-human primates (Group P); humans (Group H); avian (Group A); and fish (Group F). Known ORFs were concatenated for total codon analyses of LT-Ag. Accordingly, we performed the analysis using CDS regions in the form of the complement (join, codon start = 1) of LT-Ag from PyV reference sequences. Accession numbers are given in Table 2. To identify the domain regions contained in each *LT-Ag* gene CDS and extract the corresponding sequences, the amino acid sequence encoding each gene was scanned through PROSITE (https://prosite.expasy.org/), and the ScanProsite results were obtained in addition to ProRule-based predicted intra-domain features. The sequence information of the corresponding region was extracted and used for analysis. PROSITE provides predicted results and related information regarding protein domains, families, and functional sites through ProRule, a collection of rules based on profiles and patterns. Therefore, in this study, the sequence information of 54 DnaJ domains (PROSITE entry: PS50076) and 86 superfamily 3 helicases of DNA virus domains (PROSITE entry: PS51206), along with 86 complete gene sequences, was used for analysis (Table 3). Java programming was performed for LXCXE motif and sequence extraction and processing.

**Table 2:** Description of sequence data used in this study

No.	ICTV Taxonomy		NCBI Reference Sequence							
	Virus name	Abbr.	Accession No.	Host species	Isolation source	Country	Year	bp	Group (host)	Ref.
1	bat polyomavirus 4a	BatPyV4a	NC_038556.1	*Artibeus planirostris*	spleen	French Guiana	2011	5187	M	[24]
2	*Ateles paniscus* polyomavirus 1	ApanPyV1	NC_019853.1	*Ateles paniscus*	NA	Germany	NA	5273	P	[25]
3	bat polyomavirus 5b1	BatPyV5b-1	NC_026767.1	*Pteropus vampyrus*	spleen	Indonesia	2012	5047	M	[26]
4	bat polyomavirus 5a	BatPyV5a	NC_026768.1	*Dobsonia moluccensis*	spleen	Indonesia	2012	5075	M	[26]
5	Bornean orang-utan polyomavirus	OraPyV-Bor	NC_013439.1	*Pongo pygmaeus*	blood	NA	NA	5168	P	[27]
6	Cardioderma polyomavirus	CardiodermaPyV	NC_020067.1	*Cardioderma cor*	rectal swab	Kenya	2006	5372	M	[28]
7	bat polyomavirus 4b	BatPyV4b	NC_028120.1	*Carollia perspicillata*	spleen	French Guiana	2011	5352	M	[24]
8	chimpanzee polyomavirus	ChPyV	NC_014743.1	*Pan troglodytes verus*	blood	NA	NA	5086	P	[29]
9	vervet monkey polyomavirus 1	VmPyV1	NC_019844.1	*Chlorocebus pygerythrus*	spleen	Zambia	2009	5157	P	[30]
10	vervet monkey polyomavirus 3	VmPyV3	NC_025898.1	*Chlorocebus pygerythrus*	spleen	Zambia	2009	5055	P	[30]
11	Eidolon polyomavirus 1	EidolonPyV	NC_020068.1	*Eidolon helvum*	rectal swab	Kenya	2009	5294	M	[28]
12	*Gorilla gorilla* gorilla polyomavirus 1	GgorgPyV1	NC_025380.1	*Gorilla gorilla gorilla*	NA	Congo Republic	2008	5300	P	[31]
13	Human polyomavirus 9	HPyV9	NC_015150.1	*Homo sapiens*	NA	Germany	2009	5026	H	[32]
14	Human polyomavirus 12	HPyV12	NC_020890.1	*Homo sapiens*	NA	Germany	2007	5033	H	[33]
15	*Macaca fascicularis* polyomavirus 1	MfasPyV1	NC_019851.1	*Macaca fascicularis*	NA	Germany	NA	5087	P	[25]
16	Merkel cell polyomavirus	MCPyV	NC_010277.2	*Homo sapiens*	skin	USA	2009	5387	H	[16]
17	hamster polyomavirus	HaPyV	NC_001663.2	*Mesocricetus auratus* strain Z3	NA	Germany	1967	5372	M	[34]
18	bat polyomavirus 3b	BatPyV3b	NC_028123.1	*Molossus molossus*	spleen	French Guiana	2011	4903	M	[24]
19	mouse polyomavirus	MPyV	NC_001515.2	*Mus musculus*	NA	NA	NA	5307	M	NA
20	New Jersey polyomavirus	NJPyV	NC_024118.1	*Homo sapiens*	bicep muscle	USA	2013	5108	H	[35]
21	Otomops polyomavirus 2	OtomopsPyV	NC_020066.1	*Otomops martiensseni*	rectal swab	Kenya	2006	4914	M	[28]
22	Otomops polyomavirus 1	OtomopsPyV1	NC_020071.1	*Otomops martiensseni*	rectal swab	Kenya	2006	5176	M	[28]
23	Pan troglodytes verus polyomavirus 2a	PtrovPyV2a	NC_025370.1	*Pan troglodytes verus*	NA	Cote d’Ivoire	2010	5309	P	[31]
24	Pan troglodytes verus polyomavirus 3	PtrovPyV3	NC_019855.1	*Pan troglodytes verus*	NA	Cote d’Ivoire	NA	5333	P	[25]
25	Pan troglodytes verus polyomavirus 4	PtrovPyV4	NC_019856.1	*Pan troglodytes verus*	NA	Cote d’Ivoire	NA	5349	P	[25]
26	Pan troglodytes verus polyomavirus 5	PtrovPyV5	NC_019857.1	*Pan troglodytes verus*	NA	Cote d’Ivoire	NA	4994	P	[25]
27	Pan troglodytes schweinfurthii polyomavirus 2	PtrosPyV2	NC_019858.1	*Pan troglodytes schweinfurthii*	NA	Uganda	NA	4970	P	[25]
28	Pan troglodytes verus polyomavirus 1a	PtrovPyV1a	NC_025368.1	*Pan troglodytes verus*	NA	Cote d’Ivoire	2009	5303	P	[31]
29	Piliocolobus badius polyomavirus 2	PbadPyV2	NC_039051.1	*Piliocolobus badius*	NA	Cote d’Ivoire	2005	5148	P	[36]
30	Piliocolobus rufomitratus polyomavirus 1	PrufPyV1	NC_019850.1	*Piliocolobus rufomitratus*	NA	Cote d’Ivoire	NA	5140	P	[25]
31	raccoon polyomavirus	RacPyV	NC_023845.1	raccoon	NA	USA	2011	5016	M	[37]
32	*Rattus norvegicus* polyomavirus 1	RnorPyV1	NC_027531.1	*Rattus norvegicus*	spleen	Germany	2005	5318	M	[38]
33	bat polyomavirus 3a-B0454	BatPyV3a-B0454	NC_038557.1	*Sturnira lilium*	spleen	French Guiana	2011	5058	M	[24]
34	Sumatran orang-utan polyomavirus	OraPyV-Sum	NC_028127.1	*Pongo abelii*	blood	NA	NA	5358	P	[27]
35	Trichodysplasia spinulosa-associated polyomavirus	TSPyV	NC_014361.1	*Homo sapiens*	skin	Netherlands	2009	5232	H	[15]
36	yellow baboon polyomavirus 1	YbPyV1	NC_025894.1	*Papio cynocephalus*	spleen	Zambia	2009	5064	P	[30]
37	African elephant polyomavirus 1	AelPyV1	NC_022519.1	*Loxodonta africana*	protruding ulcerated fibroma	Denmark	2011	5722	M	[39]
38	BatPyV4a	BatPyV2c	NC_038558.1	*Artibeus planirostris*	spleen	French Guiana	2011	5371	M	[24]
39	Myodes glareolus polyomavirus 1	BVPyV	NC_028117.1	*Myodes glareolus*	blood serum and body fluids	Germany	2013	5032	M	[40]
40	bat polyomavirus 6a	BatPyV6a	NC_026762.1	*Acerodon celebensis*	spleen	Indonesia	2013	5019	M	[26]
41	bat polyomavirus 6b	BatPyV6b	NC_026770.1	*Dobsonia moluccensis*	spleen	Indonesia	2012	5039	M	[26]
42	bat polyomavirus 6c	BatPyV6c	NC_026769.1	*Dobsonia moluccensis*	spleen	Indonesia	2012	5046	M	[26]
43	California sea lion polyomavirus 1	SLPyV	NC_013796.1	*Zalophus californianus*	tongue	USA	2006	5112	M	[41]
44	*Cebus albifrons* polyomavirus 1	CalbPyV1	NC_019854.2	*Cebus albifrons*	NA	Germany	NA	5013	P	[25]
45	*Cercopithecus erythrotis* polyomavirus 1	CeryPyV1	NC_025892.1	*Cercopithecus erythrotis*	NA	Cameroon	NA	5189	P	[25]
46	vervet monkey polyomavirus 2	VmPyV2	NC_025896.1	*Chlorocebus pygerythrus*	kidney	Zambia	2009	5167	P	[30]
47	*Microtus arvalis* polyomavirus 1	CVPyV	NC_028119.1	*Microtus arvalis*	blood serum and body fluids	Germany	2013	5024	M	[40]
48	bat polyomavirus 2a	BatPyV2a	NC_028122.1	*Desmodus rotundus*	spleen	French Guiana	2011	5201	M	[24]
49	equine polyomavirus	EPyV	NC_017982.1	*Equus caballus*	eye	USA	2003	4987	M	[42]
50	BK polyomavirus	BKV; BKPyV	NC_001538.1	*Homo sapiens*	NA	NA	NA	5153	H	[43]
51	KI polyomavirus	KIPyV	NC_009238.1	*Homo sapiens*	NA	NA	NA	5040	H	[44]
52	JC polyomavirus	JCV; JCPyV	NC_001699.1	*Homo sapiens*	NA	NA	NA	5130	H	[45]
53	Weddell seal polyomavirus	WsPyV	NC_032120.1	*Leptonychotes weddellii*	kidney	Antarctica	2014	5186	M	NA
54	simian virus 40	SV40	NC_001669.1	*Macaca mulatta*	NA	NA	NA	5243	P	[46]
55	Mastomys polyomavirus	MasPyV	NC_025895.1	*Mastomys natalensis*	spleen	Zambia	2009	4899	M	[47]
56	*Meles meles* polyomavirus 1	MmelPyV1	NC_026473.1	*Meles meles*	salivary gland	France	2014	5187	M	[48]
57	Miniopterus polyomavirus	MiniopterusPyV	NC_020069.1	*Miniopterus africanus*	rectal swab	Kenya	2006	5213	M	[28]
58	mouse pneumotropic virus	MPtV	NC_001505.2	*Mus musculus*	NA	NA	NA	4754	M	[49]
59	Myotis polyomavirus	MyPyV	NC_011310.1	*Myotis lucifugus*	NA	Canada	2007	5081	M	[50]
60	Pan troglodytes verus polyomavirus 8	PtrovPyV8	NC_028635.1	Western chimpanzee	colon	Netherlands	2014	5163	P	[51]
61	Pteronotus polyomavirus	PteronotusPyV	NC_020070.1	*Pteronotus davyi*	oral swab	Guatemala	2009	5136	M	[28]
62	bat polyomavirus 2b	BatPyV2b	NC_028121.1	*Pteronotus parnellii*	spleen	French Guiana	2011	5041	M	[24]
63	rat polyomavirus 2	RatPyV2	NC_032005.1	*Rattus norvegicus*	NA	USA	2016	5108	M	NA
64	*Saimiri sciureus* polyomavirus 1	SsciPyV1	NC_038559.1	*Saimiri sciureus*	NA	Germany	NA	5067	P	NA
65	squirrel monkey polyomavirus	SquiPyV	NC_009951.1	*Saimiri boliviensis*	spleen	NA	NA	5075	P	[52]
66	alpaca polyomavirus	AlPyV	NC_034251.1	*Vicugna pacos*	NA	USA	2014	5052	M	[53]
67	WU polyomavirus	WUPyV	NC_009539.1	*Homo sapiens*	NA	Australia	NA	5229	H	[54]
68	yellow baboon polyomavirus 2	YbPyV2	AB767295.2	*Papio cynocephalus*	spleen and kidney	Zambia	2009	5181	P	[30]
69	Human polyomavirus 6	HPyV6	NC_014406.1	*Homo sapiens*	skin	USA	2009	4926	H	[16]
70	Human polyomavirus 7	HPyV7	NC_014407.1	*Homo sapiens*	skin	USA	2009	4952	H	[16]
71	MW polyomavirus	MWPyV	NC_018102.1	*Homo sapiens*	stool	Malawi	2008	4927	H	[55]
72	STL polyomavirus	STLPyV	NC_020106.1	*Homo sapiens*	fecal specimen	Malawi	NA	4776	H	[56]
73	Adélie penguin polyomavirus	ADPyV	NC_026141.2	*Pygoscelis adeliae*	fecal material	Antarctica	2012	4988	A	[57]
74	budgerigar fledgling disease virus	BFDV	NC_004764.2	Falconiformes and Psittaciformes (wild birds)	NA	NA	NA	4981	A	[58]
75	butcherbird polyomavirus	Butcherbird PyV	NC_023008.1	*Cracticus torquatus*	periocular skin	Australia	2009	5084	A	[59]
76	canary polyomavirus	CaPyV	NC_017085.1	*Serinus canaria*	liver	Netherlands	2007	5421	A	[60]
77	crow polyomavirus	CpyV	NC_007922.1	*Corvus monedula*	NA	NA	2005	5079	A	[22]
78	*Erythrura gouldiae* polyomavirus 1	EgouPyV1	NC_039052.1	*Erythrura gouldiae*	liver	Poland	2014	5172	A	[61]
79	finch polyomavirus	FPyV	NC_007923.1	*Pyrrhula pyrrhula griseiventris*	NA	NA	2005	5278	A	[22]
80	goose hemorrhagic polyomavirus	GHPV	NC_004800.1	goose	NA	Germany	2001	5256	A	[62]
81	Hungarian finch polyomavirus	HunFPyV	NC_039053.1	*Lonchura maja*	kidney and liver	Hungary	2011	5284	A	[63]
82	black sea bass-associated polyomavirus 1	BassPyV1	NC_025790.1	*Centropristis striata*	NA	USA	2014	7369	F	[64]
83	bovine polyomavirus	BPyV	NC_001442.1	*Bos taurus*	kidney	NA	NA	4697	M	[65]
84	dolphin polyomavirus 1	DPyV	NC_025899.1	*Delphinus delphis*	trachea	USA	2010	5159	M	[66]
85	giant guitarfish polyomavirus	GfPyV1	NC_026244.1	*Rhynchobatus djiddensis*	skin lesion	USA	2014	3962	F	[67]
86	sharp-spined notothenia polyomavirus	SspPyV	NC_026944.1	*Trematomus pennellii*	NA	Antarctica	2013	6219	F	NA

No. 1~36: *Alphapolyomaviruses*; No. 37~68: *Betaphapolyomaviruses;* No. 69~72: *Deltapolyomaviruses*; No. 73~81: *Gammapolyomaviruses*; No. 82~86: Unassigned polyomaviruses; *NA* Not available

All 86 viruses were classified into 5 groups according to their host as follows: non-primate mammals (Group M); non-human primate (Group P); human (Group H); avian (Group A); fish (Group F)

**Table 3:** Domains and motifs of PyVs used in this study

No.	Abbr.	Accession no.	DnaJ domain			LXCXE motif			Helicase domain		
			Start	End	nt length	Start	End	a.a. sequence	Start	End	nt length
1	BatPyV4a	NC_038556.1	12	67	168	107	111	LRCDE	405	564	480
2	ApanPyV1	NC_019853.1	12	77	198	122	126	LFCNE	441	601	483
3	BatPyV5b-1	NC_026767.1	12	74	189	–	–	–	376	536	483
4	BatPyV5a	NC_026768.1	12	67	168	–	–	–	382	546	495
5	OraPyV-Bor	NC_013439.1	12	77	198	122	126	LFCDE	422	602	543
6	CardiodermaPyV	NC_020067.1	12	77	198	212	216	LYCDE	556	716	483
7	BatPyV4b	NC_028120.1	–	–	–	152	156	LLCEE	458	651	582
8	ChPyV	NC_014743.1	12	96	255	–	–	–	379	580	606
9	VmPyV1	NC_019844.1	12	80	207	107	111	LHCNE	479	640	486
10	VmPyV3	NC_025898.1	12	75	192	131	135	LFCSE	462	622	483
11	EidolonPyV	NC_020068.1	–	–	–	236	240	LRCDE	588	752	495
12	GgorgPyV1	NC_025380.1	–	–	–	200	204	LFCDE	554	714	483
13	HPyV9	NC_015150.1	12	86	225	123	127	LFCSE	446	606	483
14	HPyV12	NC_020890.1	–	–	–	–	–	–	473	635	489
15	MfasPyV1	NC_019851.1	12	86	225	125	129	LFCTE	465	665	603
16	MCPyV	NC_010277.2	–	–	–	212	216	LFCDE	567	727	483
17	HaPyV	NC_001663.2	–	–	–	130	134	LTCQE	522	682	483
18	BatPyV3b	NC_028123.1	–	–	–	107	111	LYCDE	467	630	492
19	MPyV	NC_001515.2	–	–	–	142	146	LFCYE	549	709	483
20	NJPyV	NC_024118.1	12	80	207	107	111	LHCDE	476	636	483
21	OtomopsPyV	NC_020066.1	12	92	243	107	111	LYCDE	483	643	483
22	OtomopsPyV1	NC_020071.1	–	–	–	185	189	LRCDE	520	680	483
23	PtrovPyV2a	NC_025370.1	–	–	–	200	204	LFCDE	556	716	483
24	PtrovPyV3	NC_019855.1	12	75	192	–	–	–	486	646	483
25	PtrovPyV4	NC_019856.1	12	75	192	–	–	–	489	646	474
26	PtrovPyV5	NC_019857.1	12	86	225	123	127	LFCSE	439	599	483
27	PtrosPyV2	NC_019858.1	12	85	222	108	112	LYCSE	432	632	603
28	PtrovPyV1a	NC_025368.1	–	–	–	203	207	LYCDE	558	718	483
29	PbadPyV2	NC_039051.1	12	92	243	107	111	LHCNE	476	637	486
30	PrufPyV1	NC_019850.1	12	93	246	107	111	LHCNE	476	637	486
31	RacPyV	NC_023845.1	–	–	–	167	171	LFCEE	504	685	546
32	RnorPyV1	NC_027531.1	–	–	–	128	132	LYCSE	535	698	492
33	BatPyV3a-B0454	NC_038557.1	–	–	–	107	111	LHCHE	477	637	483
34	OraPyV-Sum	NC_028127.1	12	75	192	–	–	–	489	649	483
35	TSPyV	NC_014361.1	12	77	198	122	126	LFCHE	445	605	483
36	YbPyV1	NC_025894.1	12	75	192	131	135	LFCSE	463	663	603
37	AelPyV1	NC_022519.1	–	–	–	–	–	–	400	564	495
38	BatPyV2c	NC_038558.1	–	–	–	223	227	LLCEE	559	719	483
39	BVPyV	NC_028117.1	12	67	168	146	150	LTCHE	383	574	576
40	BatPyV6a	NC_026762.1	–	–	–	84	88	LFCHE	395	557	489
41	BatPyV6b	NC_026770.1	–	–	–	98	102	LFCHE	407	570	492
42	BatPyV6c	NC_026769.1	–	–	–	100	104	LFCRE	426	587	486
43	SLPyV	NC_013796.1	12	77	198	113	117	LHCHE	397	556	480
44	CalbPyV1	NC_019854.2	–	–	–	100	104	LFCNE	410	570	483
45	CeryPyV1	NC_025892.1	12	75	192	105	109	LFCHE	402	562	483
46	VmPyV2	NC_025896.1	12	75	192	105	109	LFCHE	402	562	483
47	CVPyV	NC_028119.1	12	67	168	145	149	LSCNE	382	573	576
48	BatPyV2a	NC_028122.1	12	80	207	–	–	–	406	565	480
49	EPyV	NC_017982.1	12	86	225	105	109	LRCDE	402	562	483
50	BKPyV	NC_001538.1	12	75	192	105	109	LFCHE	402	562	483
51	KIPyV	NC_009238.1	–	–	–	108	112	LRCNE	410	572	489
52	JCPyV	NC_001699.1	12	75	192	105	109	LFCHE	401	561	483
53	WsPyV	NC_032120.1	12	77	198	113	117	LHCNE	400	561	486
54	SV40	NC_001669.1	12	75	192	103	107	LFCSE	400	560	483
55	MasPyV	NC_025895.1	–	–	–	101	105	LFCNE	414	576	489
56	MmelPyV1	NC_026473.1	12	80	207	111	115	LRCDE	365	559	585
57	MiniopterusPyV	NC_020069.1	12	75	192	103	107	LHCHE	369	560	576
58	MPtV	NC_001505.2	–	–	–	103	107	LFCNE	418	573	468
59	MyPyV	NC_011310.1	–	–	–	–	–	–	441	603	489
60	PtrovPyV8	NC_028635.1	12	75	192	105	109	LFCHE	402	562	483
61	PteronotusPyV	NC_020070.1	12	80	207	108	112	LRCDE	405	564	480
62	BatPyV2b	NC_028121.1	12	80	207	108	112	LRCDE	406	617	636
63	RatPyV2	NC_032005.1	12	79	204	178	182	LHCDE	474	634	483
64	SsciPyV1	NC_038559.1	–	–	–	101	105	LFCHE	410	572	489
65	SquiPyV	NC_009951.1	–	–	–	101	105	LFCHE	411	570	480
66	AlPyV	NC_034251.1	12	67	168	107	111	LYCNE	407	567	483
67	WUPyV	NC_009539.1	12	89	234	108	112	LRCNE	417	579	489
68	YbPyV2	AB767295.2	12	75	192	105	109	LFCHE	402	562	483
69	HPyV6	NC_014406.1	–	–	–	109	113	LYCDE	393	571	537
70	HPyV7	NC_014407.1	–	–	–	109	113	LYCTE	416	576	483
71	MWPyV	NC_018102.1	–	–	–	105	109	LSCNE	421	580	480
72	STLPyV	NC_020106.1	12	83	216	105	109	LTCNE	406	566	483
73	ADPyV	NC_026141.2	8	61	162	69	73	LYCEE	408	582	525
74	BFDV	NC_004764.2	6	82	231	–	–	–	372	532	483
75	Butcherbird PyV	NC_023008.1	8	67	180	70	74	LFCDE	410	572	489
76	CaPyV	NC_017085.1	8	61	162	67	71	LSCNE	390	550	483
77	CpyV	NC_007922.1	11	80	210	69	73	LQCEE	405	569	495
78	EgouPyV1	NC_039052.1	8	75	204	70	74	LYCEE	374	572	597
79	FPyV	NC_007923.1	6	70	195	60	64	LFCDE	382	543	486
80	GHPV	NC_004800.1	8	81	222	65	69	LFCDE	404	599	588
81	HunFPyV	NC_039053.1	6	77	216	60	64	LFCDE	382	543	486
82	BassPyV1	NC_025790.1	–	–	–	105	109	LMCGE	338	495	474
83	BPyV	NC_001442.1	10	73	192	93	97	LHCDE	391	586	588
84	DPyV	NC_025899.1	11	77	201	82	86	LYCDE	357	536	540
85	GfPyV1	NC_026244.1	–	–	–	–	–	–	348	517	510
86	SspPyV	NC_026944.1	–	–	–	–	–	–	372	529	474

ScanProsite results together with ProRule-based predicted intra-domain features were used for functional domains retained in LT-Ag of PyVs. LXCXE motifs and their encoding sequences were extracted through the JAVA programming

### Phylogenetic analysis

Multiple sequence alignments were performed for each sequence using MUSCLE, and the phylogeny was reconstructed using the maximum likelihood (ML) method based on the Tamura-Nei model [68] using MEGA 7.0.26 [69]. Bootstrap analysis [70] was carried out with 1000 replicates of the dataset to determine the robustness of the individual nodes. The reconstructed trees confirmed the phylogenetic relationships for viral sequences of the *LT-Ag* gene, DnaJ, and helicase from different host species. Based on these results, the 86 viral species were divided into five groups [non-primate mammals (Group M), non-human primates (Group P), humans (Group H), avian (Group A), and fish (Group F)]. For the purpose of this study, virus group information based on the phylogenetic relationships was considered when conducting various analyses and interpreting and discussing the results.

### Compositional analysis

The CodonW (https://sourceforge.net/projects/codonw/) and CALcal (http://genomes.urv.es/CAIcal/) programs were used to perform nucleotide composition analysis. Various nucleotide compositional properties were calculated for the sequences corresponding to the CDS of the PyV *LT-Ag* gene, DnaJ domain, and helicase domain. The frequency of each nucleotide (%A, %C, %T, and %G), GC and AT contents (%GC and %AT), each nucleotide at the third position of synonymous codons (%A3, %C3, %T3, and %G3), G + C (%GC3) and A + T contents (%AT3) at the third codon, and G + C (%GC12) and A + T mean values (%AT12) at the first and second codons were calculated. Genetic variability was analyzed by calculating the nucleotide variability of the *LT-Ag* genes and two domains in each virus group. The total number of segregating sites, total number of mutations, average number of nucleotide differences between sequences, and nucleotide diversity were estimated using DnaSP v. 5.10.01 [71].

### Effective number of codons (ENC) analysis

Analysis of the effective number of codons (ENC) was used to quantify the absolute codon usage bias in the PyV *LT-Ag* gene CDS, independent of the gene length. ENC values range from 20 to 61; 20 represents the largest codon usage bias, in which only one of the possible synonymous codons is used for the corresponding amino acid; 61 indicates no bias and means that all possible synonymous codons are used equally for the corresponding amino acid. Generally, genes are considered to have significant codon bias when the ENC value is less than 35 [72, 73].

### Parity rule 2 (PR2) analysis

Parity rule 2 (PR2) analysis is commonly used to investigate the effects of mutations and selection pressure on codon usage bias in genes. The PR2 plot positions the AT-bias [A3/(A3 + T3)] and GC-bias [G3/(G3 + C3)] at the third codon of four-codon amino acids [fourfold degenerate codon families: Ala (A), Arg (R), Gly (G), Leu (L), Pro (P), Ser (S), Thr (T), and Val (V)] of the entire genome are shown on the vertical axis (y) and horizontal axis (x), respectively. The location of the plot with both coordinates at 0.5 is A = T, G = C (PR2), indicating no bias between the effects of mutation and natural selection (replacement rate). The distance between the coordinate position (0.5, 0.5) and the plot dot, which is the center of the plot, indicates the degree and direction of the PR2 bias [74, 75].

### Neutral evolution analysis

Neutrality plots are used to evaluate the relationship between the third codon positions to reflect the role of directional mutation pressure. Consequently, the gradients of the regression lines in the neutrality plot depict the relationship between GC12s and GC3s, elucidating the evolutionary rates of directional mutation pressure–natural selection equilibrium. When the gradient of the regression line is 0 (all plot dots are located on a line parallel to the abscissa), there are no effects from directional mutation pressure. When the gradient is 1 (all plot dots are located on the diagonal), we have complete neutrality. Therefore, the regression lines of the neutrality plot can be used to determine the main factor controlling evolution by measuring the degree of neutrality [76]. DnaSP v. 5.10.01 [71] was used to calculate Tajima’s D [77], Fu and Li’s D*, and F* [78] as tests of neutrality. Tajima’s D statistic measures the departure from neutrality for all mutations in a genomic region [77] and is based on the differences between the number of segregating sites and the average number of nucleotide differences. Fu and Li’s D* test is based on the differences between the number of singletons (mutations appearing only once in the sequence) and the total number of mutations. Fu and Li’s F* test is based on the differences between the number of singletons and the average number of nucleotide differences between every pair of sequences [78, 79].

### Relative synonymous codon usage (RSCU) analysis

Relative synonymous codon usage (RSCU), a measure of the preference for the use of a synonymous codon, is defined as the ratio of the observed number of synonymous codons used to the expected value of the codon occurrence frequency [80]. In general, codons with an RSCU value greater than 1.0 are considered to have a higher preference (abundant codons), and those with an RSCU value lower than 1.0 have a lower preference (less-abundant codons). When the RSCU value is equal to 1.0, either the preference for synonymous codons is the same or the codon usage is random [81]. Specifically, a codon with an RSCU value of 1.6 or more is an over-represented codon, and a codon with an RSCU value of 0.6 or less is considered an under-represented codon (≤0.6) [82]. Using the CodonW and CAIcal programs, the RSCU values of the sequences of the 54 DnaJ domains and 86 helicase domains were calculated, along with 86 *LT-Ag* gene CDS. Comparative analysis and visualization of each group were performed using XLSTAT.

### Calculation of the codon adaptation index (CAI)

The codon adaptation index (CAI) is a quantitative measurement ranging from 0 to 1 that predicts gene expression levels based on CDS. The most frequent codons show the highest relative adaptation to the host, and sequences with a higher CAI are preferred over those with a lower CAI [83]. CAI analysis of the *LT-Ag* gene CDS was carried out using CAIcal [84], and the synonymous codon usage pattern of *Homo sapiens*, which was downloaded from the Codon Usage Database (CUD) [85], was used as the reference dataset.

### Correspondence analysis (COA)

Each group of RSCU values was analyzed using the correspondence analysis (COA) method, and the results were visualized using XLSTAT. Individual data representing the *LT-Ag* gene coding region were expressed as a vector with 59 dimensions, and we included 59 codons, excluding methionine (ATG) and tryptophan (TGG), without synonymous codons in the analysis.

### Selection pressure analysis

The number of non-synonymous substitutions per non-synonymous site (dN), the number of synonymous substitutions per synonymous site (dS), and the dN/dS ratios for the nucleotide sequences of the *LT-Ag* genes and two domains were estimated for all isolates in each virus group using MEGA 7.0.26 [69]. A gene is under positive (or diversifying) selection when the dN/dS ratio is > 1, neutral selection when dN/dS ratio = 1, and negative (or purifying) selection when the dN/dS ratio < 1.

## Results

### Sequence similarity and evolutionary relationships among PyVs

**Fig. 1:**
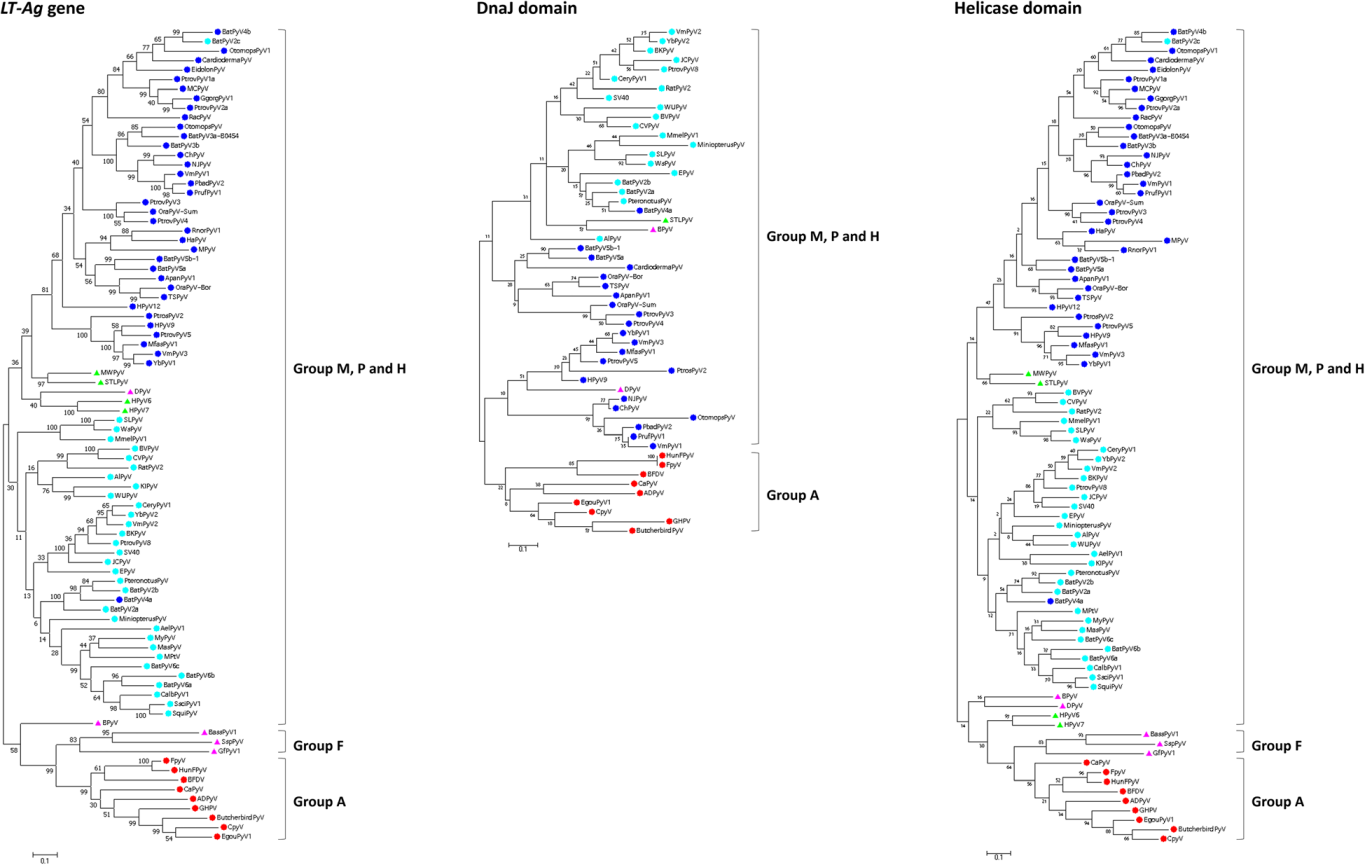
Phylogenetic trees of PyV *LT-Ag* genes. PyVs were classified according to the host species (mammal, avian, and fish) in the ML-based trees constructed using nucleotide sequences of LT-Ag coding genes, DnaJ domains, and helicase domains (*Alphapolyomaviruses* []; *Betaapolyomaviruses* []; *Deltapolyomaviruses* []; *Gammapolyomaviruses* []; unassigned [])

Phylogenetic analyses using the *LT-Ag* gene, DnaJ domain, and helicase domain revealed that, except for two bat viruses, *Alphapolyomavirus* and *Betapolyomavirus* were grouped independently, and *Gammapolyomavirus* formed a separate cluster. *Deltapolyomavirus* and the unassigned viruses clustered together or were independent in all of the trees. Thus, except for some exceptional cases [bat PyV 2c (BatPyV2c), bat PyV 4a (BatPyV4a), and DPyV] in the ML-based tree, the viruses were generally grouped by genes. When the clustering pattern per host was examined, Groups M, P, and H formed a large cluster. In other trees, except for the DnaJ domain-based tree for which domain information was lacking (Group F was not included in the analysis), Group A (avian viruses) and Group F (fish viruses) were grouped independently (Fig. 1).

### Compositional properties of *LT-Ag* genes

**Fig. 2:**
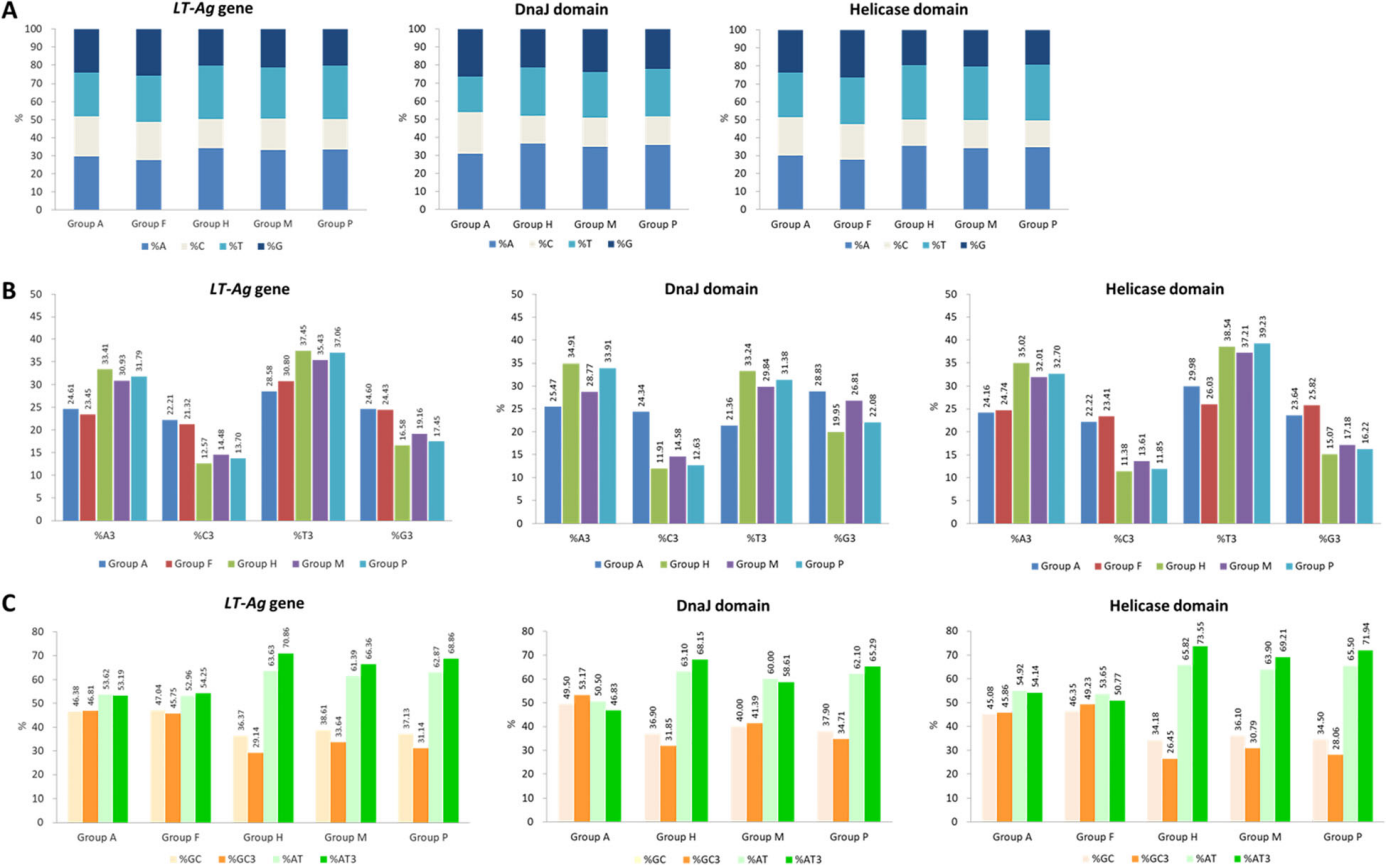
Compositional features of nucleotide sequences of LT-Ag coding genes, DnaJ domains, and helicase domains. **a** Nucleotide distribution of A, C, U, and G. **b** Distribution frequency calculated only for the third codon base. **c** GC and AT content at all codon positions (GC% and AT%) and at the third position (GC3s and AT3s)

To confirm the effect of differences in composition on the codon usage patterns observed in 86 PyV species isolated from different hosts, we analyzed the nucleotide compositions of the complete sequences of the *LT-Ag* genes, as well as those of the DnaJ domain and helicase domain regions of the LT-Ag protein, in each virus (Table 4). These domains play particularly important roles in the biochemical function of LT-Ag and are relatively well conserved in various PyV species compared to other domain regions. Thus, it is possible to extract more accurate homologous sequences based on the protein sequence pattern and profile information using these domains. Hence, these became the subjects of this analysis. After analyzing the mean composition of each group (%), nucleotide A was the highest in all groups, and C was lowest in all sequences except for the DnaJ domain CDS of Group A (Fig. 2). In the nucleotides observed at the third position of the synonymous codons (A3, T3, G3, and C3), G3 was higher than C3. T3 was higher than A3 in all groups except Group A, H, and P of the DnaJ domain. In all analyzed sequences, the GC and GC3 values were significantly higher in Groups A and F (> 45), and Groups H, M, and P exhibited high AT and AT3 values (> 60). In particular, group H viruses had significantly higher AT3 values (> 70). According to the nucleotide frequency at the third position of the codon, all sequences except the DnaJ domain CDS of avian PyVs belonging to Group A were AT-rich, but at the individual nucleotide level, G and A were dominant over C and T. In previous studies, the GC values for the entire genomes of JCPyV, BKPyV, SV40, budgerigar fledgling disease virus (BFDV), MPyV, goose hemorrhagic PyV (GHPyV), and bovine PyV (BPyV) were 0.41, 0.41, 0.42, 0.5, 0.48, 0.42, and 0.42, respectively, and the GC3 values were 0.3, 0.28, 0.31, 0.45, 0.42, 0.43, and 0.33, respectively [86]. Based on the LT-Ag CDS results for the above viruses, the %GC values of the corresponding virus were 38.12, 35.82, 37.85, 46.44, 46.57, 44.43, and 38.55, respectively, and the %GC3 values were 33.82, 28.16, 34.27, 47.67, 44.06, 44.11, and 33.06, respectively. As in previous studies using whole genome sequences, the GC and GC3 values of the bird PyV in the *LT-Ag* gene were higher than those of the mammalian PyV.

**Table 4:**
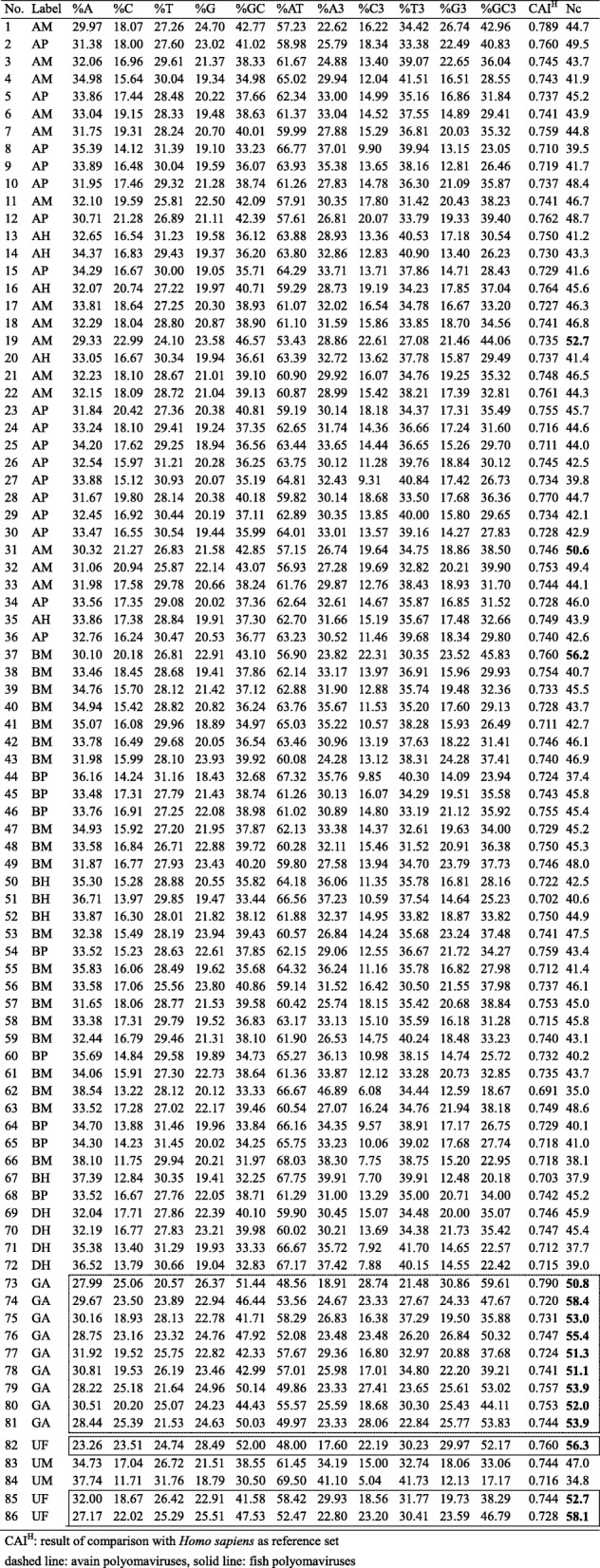
Nucleotide compositions of the *LT-Ag* genes of 86 polyomaviruses

CAI^H^: result of comparison with *Homo sapiens* as reference set

dashed line: avain polyomaviruses, solid line: fish polyomaviruses

### Codon usage patterns in the *LT-Ag* genes from different hosts

**Fig. 3:**
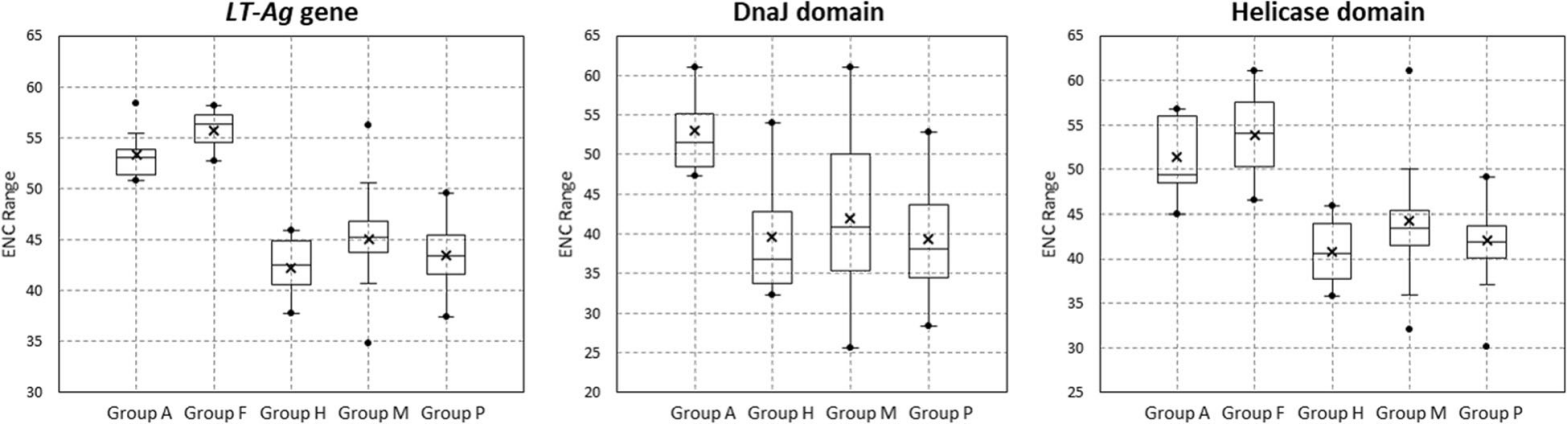
The range of ENC values of the *LT-Ag* genes and two functional domains. The cross (×) indicates the mean ENC value, and the dot (•) indicates the minimum/maximum ENC value of the *LT-Ag* genes and two domains within LT-Ag. Each group, which we classified by host, was composed of 9 (Group A), 3 (Group F), 13 (Group H), 36 (Group M), and 25 (Group P) nucleotide sequence data of *LT-Ag* genes and helicase domains. DnaJ domains were not identified in 32 protein sequences, including 3 fish PyVs; thus, a total of 54 sequence data were used for the analysis

The ENC values were calculated to estimate the magnitude of the codon usage bias in the LT-Ag sequences of the PyVs. A mean value of 45.4 ± 4.9 was confirmed for all *LT-Ag* gene sequences analyzed. The lowest ENC value was observed in dolphin PyV 1 (DPyV) (34.8), and the highest value was observed in BFDV (58.4). Groups A and F viruses had ENC ranges of 50.8–58.4 and 52.7–58.1, respectively. The mean ENC values of Groups H, M, and P viruses were 42.254, 45.078, and 43.520, respectively, significantly lower than those of Groups A and F (53.311 and 55.700, respectively). Thus, the sequence compositions in the *LT-Ag* gene according to host species had higher ENC values (> 50) in avian PyV and fish PyV than in mammalian PyV (Groups M, P, and H), implying that the codon diversity was greater in the LT-Ag CDS region of Groups A and F viruses. A similar ENC range pattern was observed in both domains. In the DnaJ domain, Group A viruses had an ENC range of 47.26–61.0. The mean ENC values of Groups H, M, and P viruses were 39.5, 42.0, and 39.3, respectively, significantly lower than the mean ENC value of Group A (53.0). In the helicase domain, Groups A and F viruses had ENC ranges of 44.94–56.81 and 46.53–61.0, respectively. The mean ENC values of Groups H, M, and P viruses were 40.8, 44.3, and 42.0, respectively, which were significantly lower than those of Groups A and F (51.5 and 53.9, respectively). These results indicate that host-specific ENC value distribution characteristics were present in the *LT-Ag* gene and the CDS of the domain regions contained in the *LT-Ag* gene. Whereas avian PyV and fish PyV included significant codon diversity, mammalian viruses belonging to Groups M, P, and H exhibited conservative and host-specific evolution of codon usage bias (Table 4, Fig. 3). Genetic variability, which was estimated by measuring the average number of pairwise nucleotide differences (k) and nucleotide diversity (π), was highest for the *LT-Ag* gene (k = 910.333, π = 0.54939) and helicase domain (k = 210, π = 0.46358) in Group F (Table 5).

**Table 5:** Nucleotide diversity, selection pressure, and neutrality tests of the *LT-Ag* genes and two domains of the PyV groups

				Genetic variability				Neutrality tests			Selection pressure
Region	Group	m	n	S	η	k	π	Tajima’s D	Fu and Li’s D	Fu and Li’s F	dN/dS
LT-Ag	All	86	944	837	2129	418.245	0.44306	−0.04390^ns^	1.45113^ns^	0.96702^ns^	2.163
	Group A	9	1725	1283	2383	737.889	0.42776	−0.82814^ns^	0.0858^ns^	−0.15345^ns^	0.282
	Group F	3	1657	1209	1522	910.333	0.54939	NA	NA	NA	0.684
	Group H	13	1648	1336	2725	725.192	0.44004	−0.80590^ns^	0.16114^ns^	−0.11521^ns^	1.673
	Group M	36	1404	1205	2813	615.989	0.43874	−0.35097^ns^	0.89680^ns^	0.54139^ns^	0.523
	Group P	25	1602	1268	2653	666.147	0.41582	− 0.20916^ns^	0.88010^ns^	0.62234^ns^	0.318
DnaJ domain	All	54	160	144	352	71.204	0.44503	−0.28170^ns^	1.14715^ns^	0.71186^ns^	0.261
	Group A	9	162	119	214	68.083	0.42027	−0.70347^ns^	0.14282^ns^	−0.07065^ns^	0.298
	Group H	7	192	146	237	82.143	0.42783	−0.88626^ns^	−0.18339^ns^	− 0.37879^ns^	0.417
	Group M	19	162	136	277	63.474	0.39181	−0.83778^ns^	0.31536^ns^	−0.03513^ns^	0.289
	Group P	19	192	153	291	78.585	0.4093	−0.23632^ns^	0.71490^ns^	0.50101^ns^	0.262
Helicase domain	All	86	424	348	827	165.867	0.3912	0.02756^ns^	1.22733^ns^	0.85387^ns^	0.316
	Group A	9	471	288	499	159.361	0.33835	−0.68870^ns^	0.11803^ns^	−0.08782^ns^	0.150
	Group F	3	453	285	345	210	0.46358	NA	NA	NA	0.379
	Group H	13	477	326	632	174.667	0.36618	−0.65740^ns^	0.21440^ns^	−0.02451^ns^	0.260
	Group M	36	447	346	738	170.876	0.38227	−0.15171^ns^	0.86494^ns^	0.60171^ns^	0.503
	Group P	25	471	317	619	161.56	0.34301	−0.05815^ns^	0.97206^ns^	0.75361^ns^	0.142

m, number of sequences used; n, total number of sites (excluding sites with gaps/missing data); S, number of segregating sites; η, total number of mutations; k, average number of pairwise nucleotide differences; π, nucleotide diversity; dS, average number of synonymous substitutions per site; dN, average number of non-synonymous substitutions per site; NA, not available due to limited sequences for analysis of the gene-specific sequence dataset; ns, not significant

**Fig. 4:**
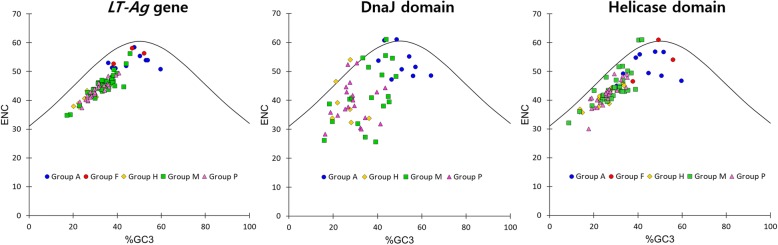
The relationship between ENC and GC3 (N_C_ plot). ENC were plotted against GC content at the third codon position. The expected ENC from GC3 are shown as a solid line

The NC plot showing the relationship between ENC and GC3 revealed that the results from excluding eight DnaJ domains and three helicase domain CDS, while including the entire *LT-Ag* gene CDS were plotted under the expected ENC curve, suggesting that the codon usage was biased. This pattern was observed overall, regardless of group. However, in the *LT-Ag* gene sequence analysis, Groups A and F viruses exhibited more diverse codon usage, as they were located closer to the expected ENC curve. However, Groups M, P, and H had relatively more biased codon usage (Fig. 4). This codon usage pattern was consistent with the characteristics of the avian virus, which is known to have a broad host range, as opposed to the mammalian virus, with a narrow host range [7].

**Fig. 5:**
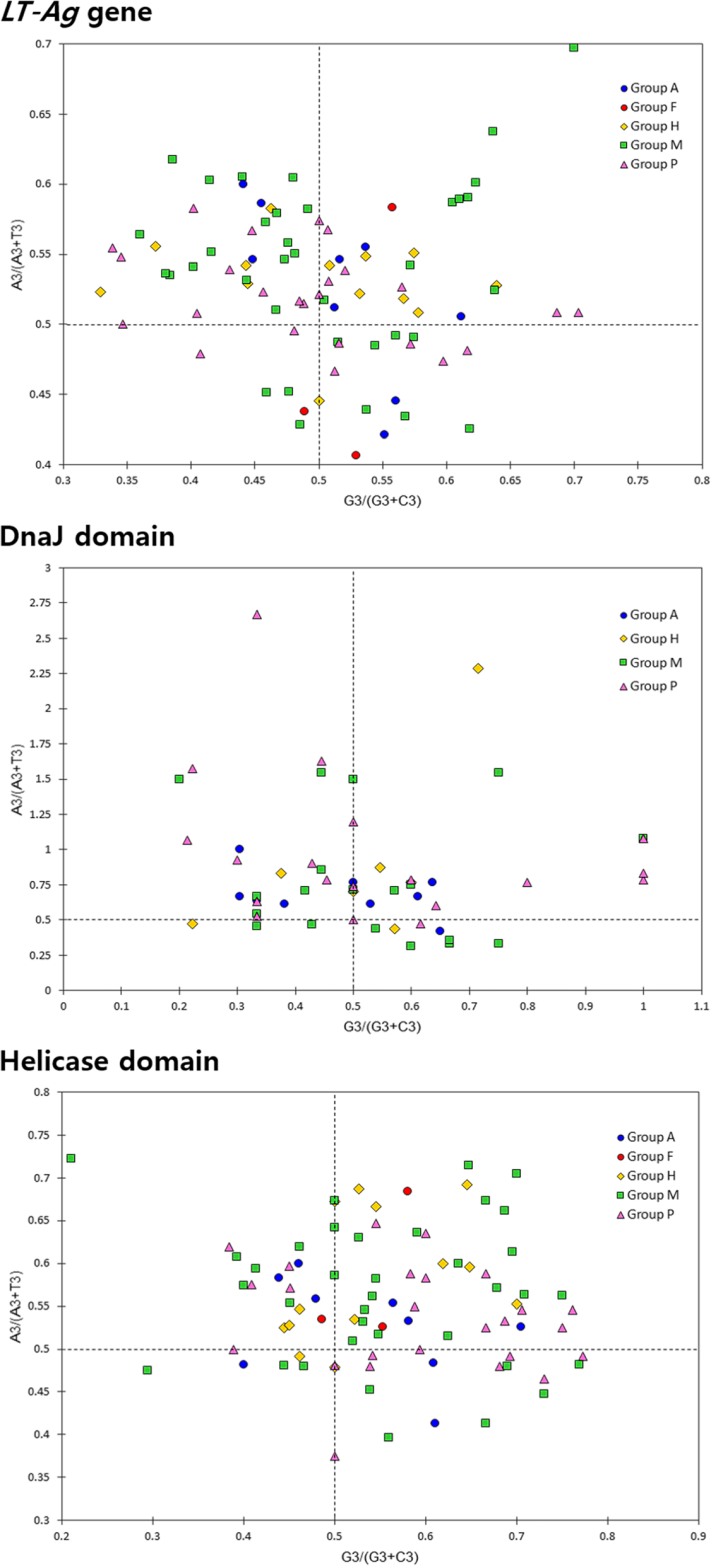
PR2-bias plot analysis. A3/(A3 + T3) were plotted against G3/(G3 + C3). The A3 content is greater than T3, and the G3 content is greater than C3 in CDS of *LT-Ag* genes, DnaJ domains, and helicase domains from different host species. These *LT-Ag* genes and their retained domains prefer to use the T-end and G-end codons

PR2 and neutrality analyses were performed to investigate the effects of mutation pressure and natural selection on codon usage patterns of LT-Ag CDS of PyVs. After analyzing the relationship between AT and GC contents, A was used at the third codon position of 65 fourfold degenerate codon families of 86 gene sequences at a frequency higher than or equal to T; in the fourfold degenerate codon families of 45 gene sequences, G was used at a frequency equal to or greater than C. In the DnaJ domain, A was used at the third codon position of 43 fourfold degenerate codon families of 54 gene sequences at a frequency higher than or equal to T, and in the fourfold degenerate codon families of 31 gene sequences, G was used at a frequency greater than or equal to C. In the helicase domain, A was used at the third codon position of 64 fourfold degenerate codon families of 86 sequences at a frequency higher than or equal to T, and in the fourfold degenerate codon families of 63 gene sequences, G was used at a frequency equal to or greater than C. When the distances and directions of all plot dots from the plot coordinate (0.5, 0.5) were examined, there were no significant differences between groups, and various distance distributions and similar directionality (T → A) were detected. Therefore, the bias shown in the PR2 plot results from the difference in the usage frequencies of T and A, which is generally shown in the fourfold degenerate codon families of the sequences encoding the *LT-Ag* genes of the PyVs and the domains contained therein, rather than differences between the groups. Unequal use of these nucleotides may imply the overlapping effect of natural selection and mutation pressure on codon selection in the corresponding gene sequences (Fig. 5). Negative values of Tajima’s D, Fu and Li’s D*, and Fu and Li’s F* were obtained for the DnaJ domain in Group H, indicating an excess of low-frequency polymorphisms caused by background selection, genetic hitchhiking, or population expansions [79, 87, 88]. The values of Tajima’s D, Fu and Li’s D*, and Fu and Li’s F* for the helicase domain in the overall population were positive, which arose from an excess of intermediate-frequency alleles and can result from population bottlenecks, structure, or balancing selection [87]. However, the *P*-values for Tajima’s D, Fu and Li’s D*, and Fu and Li’s F* tests were not significant (*P* > 0.10) in all cases (Table 5), indicating that the results were less convincing; it is also plausible that purifying selection is acting on each of the viral groups. It was impossible to do these statistical tests for the DnaJ domain in Group F, as the analysis using DnaSP software requires at least four sequences [71].

**Fig. 6:**
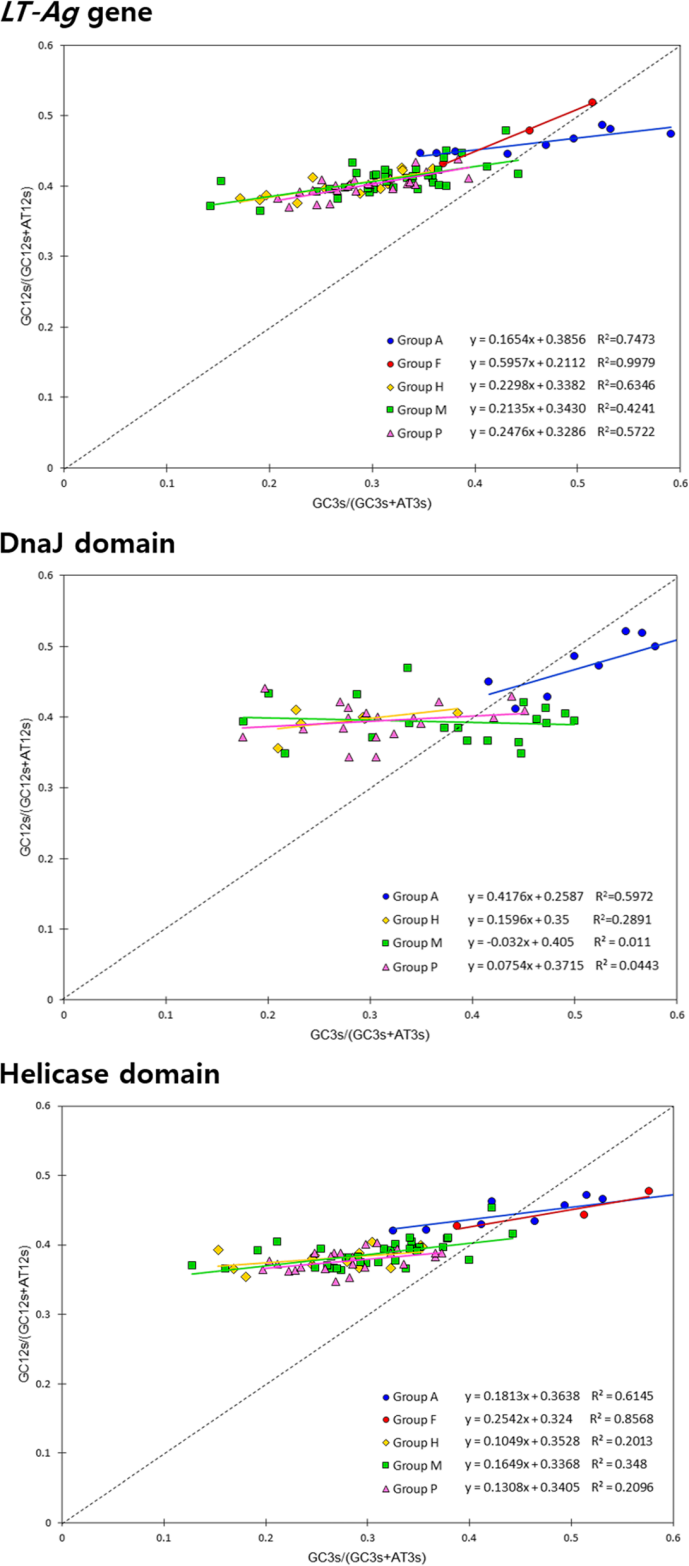
Neutrality plot of GC12 vs. GC3. GC12 were plotted against GC3. GC12 is the ordinate, and GC3 is the abscissa, so each point in the figure represents one *LT-Ag* gene from a different host organism. The neutrality plotting results for *LT-Ag* genes show that the distribution of GC12 is relatively concentrated, GC3 is during 0.171 (*Delphinus delphis* [short-beaked common dolphin]) to 0.596 (*Pygoscelis adeliae* [Adélie penguin]). Neutrality plotting results for two functional domains also show that the distribution of GC12 is relatively concentrated, while GC3 is incompactly dispersed in the range of 0.175 (*Pongo pygmaeus* [Bornean orangutan]) to 0.646 (*Pygoscelis adeliae* [Adélie penguin]) for DnaJ domains and 0.128 (*Delphinus delphis* [short-beaked common dolphin]) to 0.606 (*Pygoscelis adeliae* [Adélie penguin]) for helicase domains

In terms of the evolution of synonymous codon usage, mutation pressure either increases or decreases the GC content, and the GC content (GC3) at the third codon position expresses the most neutral nucleotides that make an important contribution to directional mutation pressure [76]. Thus, the effect of directional mutation and natural selection on the codon usage pattern of the PyV’s *LT-Ag* gene CDS isolated from different host species and two functional domains contained in the gene was estimated based on the neutrality plot. Neutrality analysis also confirmed that mutation pressure and natural selection both affected the codon usage bias of the *LT-Ag* gene CDS. The analyzed genes showed a narrow GC12 distribution and a wide GC3 distribution, indicating a significant correlation (*r* = 0.715, *p* < 0.0001). This may indicate high mutation bias or highly variable GC contents in the corresponding genes. When comparing the gradients of the regression lines for each group, Group F had the largest regression slope of 0.5957, followed by Groups P (0.2476), H (0.2298), M (0.2135), and A (0.1654). This indicates that the relative neutrality (directional mutation pressure) of the viruses belonging to each group was 59.57, 24.76, 22.98, 21.35, and 16.54%, respectively. Therefore, the contribution of natural selection to the codon usage pattern of each group was higher in the order of Groups A (83.46%), M (78.65%), H (77.02%), and P (75.24%). Group F was less affected by natural selection than the other groups (40.43%). A comparison of the gradients of the regression lines of all groups based on our neutrality analysis of the helicase domain revealed that the contribution of natural selection to the codon usage pattern of each group was, in descending order, Groups H (89.51%), P (86.92%), M (83.51%), and A (81.87%). Group F was less affected by natural selection than the other groups were (74.58%). In the case of the DnaJ domain, natural selection had a relatively low effect on Group A (58.24%), whereas its effect on other groups (Groups H, M, and P) was 80% or higher. Thus, the effect of the relative neutrality (directional mutation pressure) was found to be large (Fig. 6).

### Variation in RSCU value and codon usage preference

**Fig. 7:**
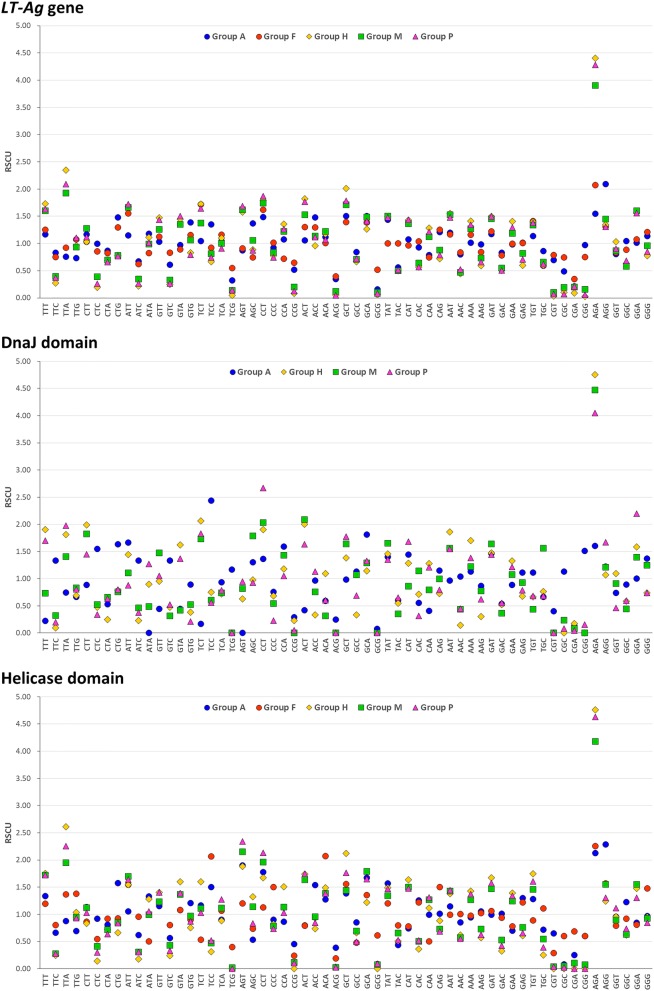
RSCU analysis of PyVs. There is variation in the differences between the codon preferences of the five groups in terms of the *LT-Ag* genes. We can see that there are relatively large differences among groups in the RSCU values of specific codons, such as codon AGA(arg) and TTA(leu)

**Fig. 8:**
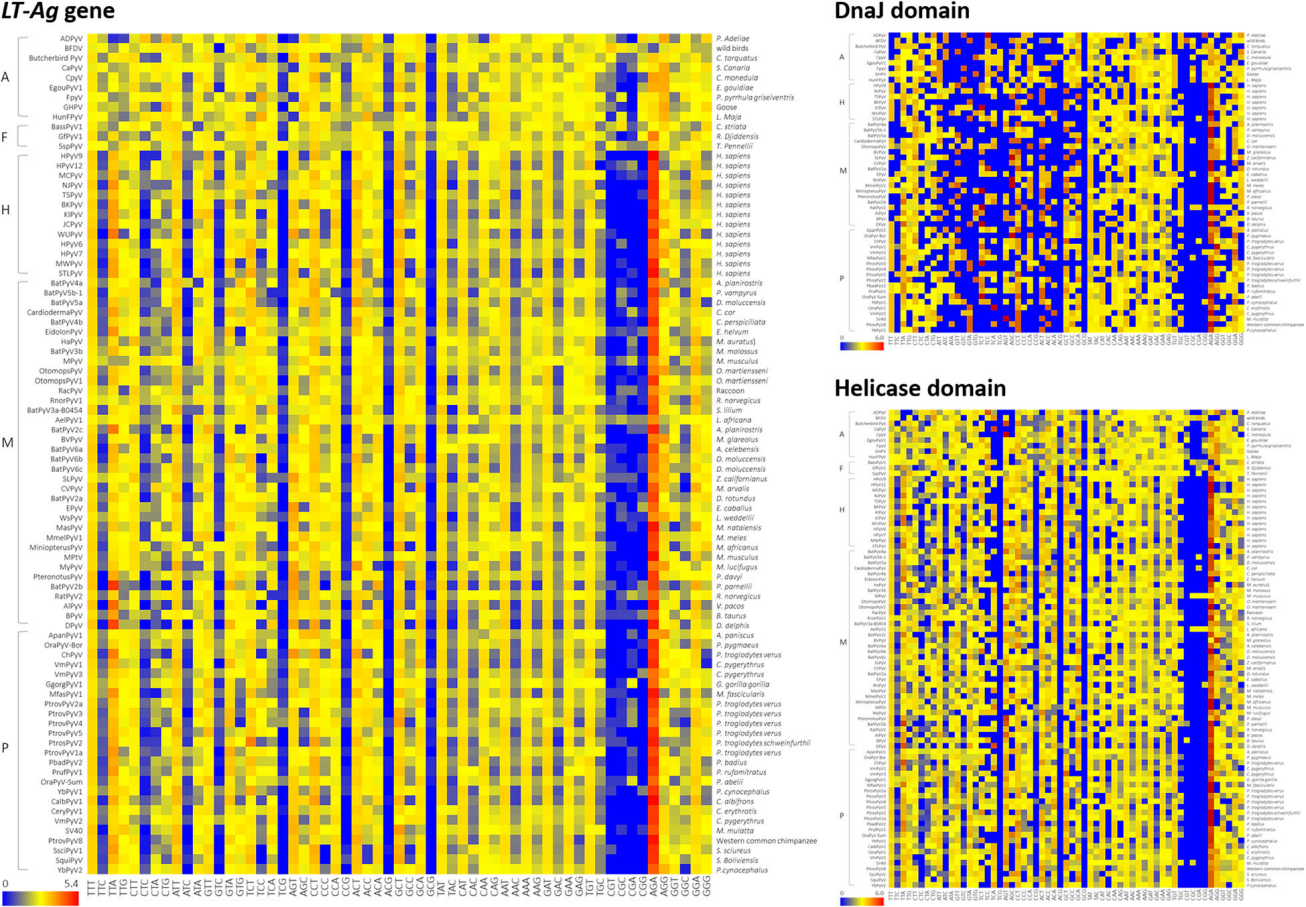
Difference in RSCU values of 86 PyVs. Respective RSCU of the 86 LT-Ag coding genes, 54 DnaJ domain coding sequences, and 86 helicase coding sequences. All RSCU values are shown in the chromaticity diagram via chromaticity co-ordinates. The chrominance difference enables visual comparison of large data sets with various host species

We calculated the RSCU values reflecting the codon preference in the *LT-Ag* genes of PyVs and analyzed their distribution pattern by group (Fig. 7) to compare them in terms of their host species (Fig. 8). First, the total mean RSCU values of the *LT-Ag* gene CDS in 86 species were calculated. The mean RSCU values for TTA (leu), ATT (ile), CCT (pro), GCT (ala), and AGA (arg) were 1.88, 1.62, 1.76, 1.74, and 3.78, respectively. Thus, they were over-represented codons. When the distribution pattern for each group was examined, the differences in codon usage preference among the mammalian viruses belonging to Groups H, M, and P were not significant. The difference between Groups A and F and the three groups of avian and fish viruses was relatively large. When the mean RSCU values of each group were compared, Groups H, M, and P had mean RSCU values of 1.6 or higher in codon TTT (phe), TTA (leu), ATT (ile), and GCT (ala), differing from Groups A and F. Codon AGA (arg) exhibited the largest difference in codon usage preference among the groups, and the mean RSCU value for each group was 1.55 (Froup A), 2.07 (F), 4.40 (H), 3.90 (M), and 4.28 (P). The color distribution according to the group or host species in Fig. 8 confirms such differences. Based on the analysis of each domain, the mean RSCU values of CCT (pro), ACT (thr), AGA (arg), and GGA (gly) were 2.13, 1.64, 3.88, and 1.64, respectively, in terms of the 54 DnaJ domain CDS. Thus, they were over-represented codons. When we compared the mean RSCU values of each group, Groups H, M, and P exhibited values of 1.6 or higher in codon TCT (ser), CCT (pro), and ACT (thr), showing differences from Group A. The total mean RSCU values for 86 helicase domain CDS were 1.66, 2.00, 2.09, 1.95, 1.70, and 4.12 for TTT (phe), TTA (leu), AGT (ser), CCT (pro), GCT (ala), and AGA (arg), respectively, indicating over-represented codons. When the mean RSCU values of the groups were compared, Groups H, M, and P had values greater than 1.6 in codon TTT (phe), TTA (leu), and ACT (thr), differing from Groups A and F. The codons AGT (ser) and CCT (pro) had values greater than 1.6 in all groups except Group F. Similar to the *LT-Ag* gene CDS, the greatest difference in codon usage preference between the groups was detected in the case of codon AGA (arg) in the two functional domains. The mean RSCU values for each group were 1.6 (Group A), 4.76 (H), 4.47 (M), and 4.05 (P) in the DnaJ domain and 2.13 (Group A), 2.26 (F), 4.76 (H), 4.18 (M), and 4.63 (P) in the helicase domain.

**Fig. 9:**
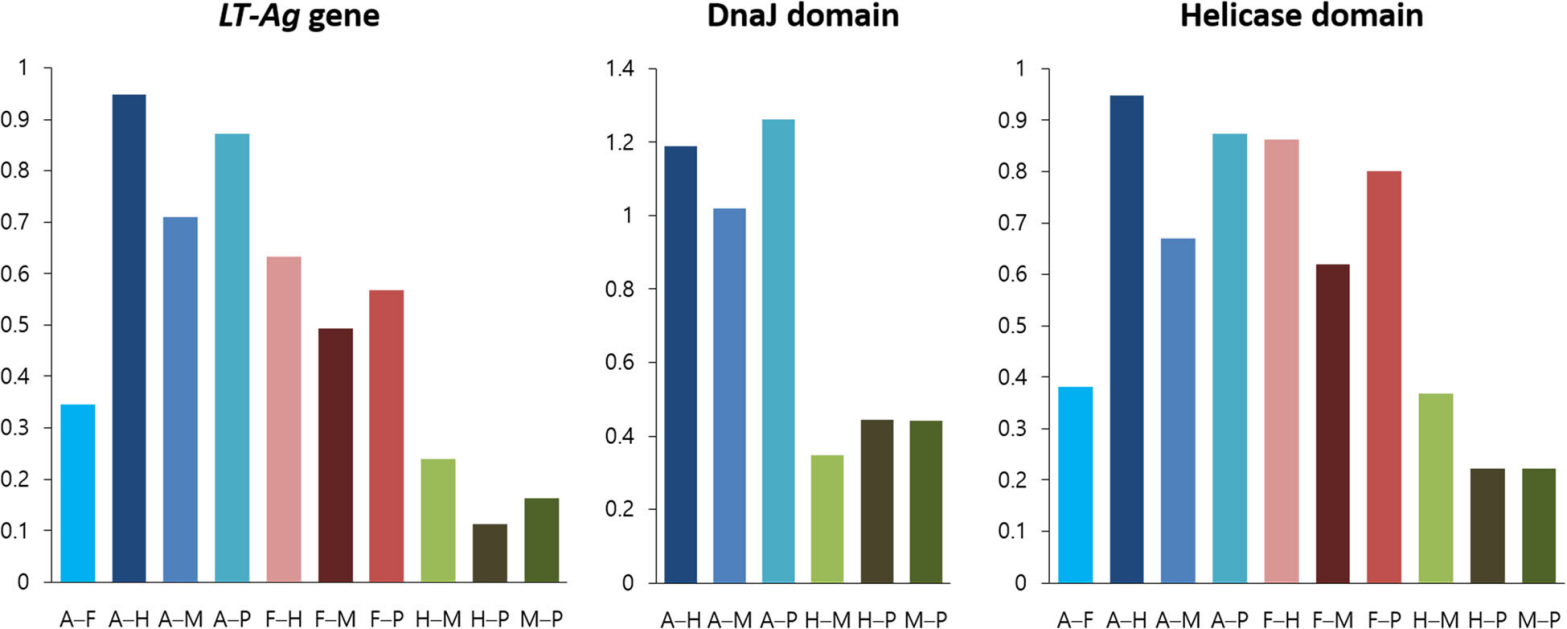
Mean RSCU distances of the host pairs calculated from the RSCU values for the abundant codons (RSCU ≥1.6) in the *LT-Ag* genes and two domains of PyVs

A preference for a particular codon is a common evolutionary phenomenon, reflecting the evolution of the biological group and carrying important meaning as a tool for explaining basic biological phenomena at the molecular level. RSCU analysis is one of the most important methods for analyzing synonymous codons in various organisms, including viruses. As shown in Fig. 7 and Fig. 8, the RSCU values of 86 *LT-Ag* genes differed by group and host, and there were differences in preference for codon usage. In Table 6 and Fig. 9, the results of comparing the mean RSCU and codon frequencies between different viral groups with their respective host species are seen more clearly. Notably, the greatest difference in codon usage preference between genes and groups was detected in codon AGA (arg) of all datasets. The CAI was calculated to compare the adaptability of synonymous codon usage. In this study, the CAI value of *H. sapiens* was used as the reference dataset. The range of the total value was 0.690–0.790, and the mean ± standard deviation was 0.74 ± 0.02. The CAI values did not vary significantly between groups, and PyVs derived from various host species generally had high similarity to the reference data in terms of both codon usage pattern and expression level. Thus, regardless of the host species, they showed relatively high adaptability in human hosts.

**Table 6:** RSCU distances of the host pairs calculated from the RSCU values for the abundant codons (RSCU ≥1.6) in the *LT-Ag* genes and two domains of PyVs

Region	Host pairs	RSCU distances witin host pairs for abundant codons (RSCU≥1.6)									
		TTT	TTA	ATT	TCT	CCT	ACT	GCT	AGA	AGG	Avg.
LT-Ag	A–F	0.082	0.165^b^	0.406	0.676	0.134	0.244	0.111	0.521	0.780	0.346
	A–H	0.558^a^	1.593^a^	0.522	0.680^a^	0.301	0.764^a^	0.507	2.855^a^	0.749	0.948
	A–M	0.435	1.169	0.512	0.334	0.257	0.471	0.204	2.355	0.645	0.709
	A–P	0.460	1.335	0.572^a^	0.601	0.385^a^	0.707	0.279	2.731	0.785^a^	0.873
	F–H	0.477	1.428	0.117	0.004^b^	0.167	0.520	0.618^a^	2.335	0.032	0.633
	F–M	0.353	1.004	0.107	0.342	0.123	0.227	0.315	1.834	0.135	0.493
	F–P	0.378	1.170	0.166	0.074	0.251	0.463	0.389	2.210	0.005^b^	0.567
	H–M	0.123	0.424	0.010^b^	0.346	0.044^b^	0.293	0.303	0.501	0.104	0.239
	H–P	0.098	0.259	0.050	0.079	0.083	0.057^b^	0.228	0.125^b^	0.036	0.113
	M–P	0.025^b^	0.166	0.060	0.267	0.127	0.236	0.075^b^	0.376	0.140	0.164
DnaJ	A–H	1.682^a^	1.064	0.224^b^	1.896^a^	0.542	1.580	0.395	3.157^a^	0.151	1.188
	A–M	0.511	0.662	0.561	1.566	0.669	1.668^a^	0.651	2.874	0.012^b^	1.019
	A–P	1.476	1.230^a^	0.788^a^	1.664	1.304^a^	1.212	0.786^a^	2.447	0.449	1.262
	H–M	1.171	0.402	0.338	0.330	0.127^b^	0.088^b^	0.256	0.283^b^	0.139	0.348
	H–P	0.207^b^	0.166^b^	0.564	0.232	0.762	0.368	0.391	0.711	0.600^a^	0.444
	M–P	0.965	0.568	0.226	0.098^b^	0.635	0.456	0.135^b^	0.427	0.461	0.441
Helicase	A–F	0.142	0.494	0.489	0.633	0.653	0.003^b^	0.168	0.128	0.716	0.381
	A–H	0.416	1.739^a^	0.492	0.435	0.106^b^	0.946	0.735^a^	2.628^a^	1.044^a^	0.949
	A–M	0.386	1.074	0.647^a^	0.059	0.184	0.837	0.053^b^	2.052	0.737	0.670
	A–P	0.363	1.349	0.594	0.049	0.445	1.013	0.599	2.502	0.949	0.873
	F–H	0.558^a^	1.245	0.002^b^	1.069^a^	0.546	0.949	0.568	2.500	0.328	0.863
	F–M	0.528	0.580	0.158	0.574	0.836	0.840	0.115	1.924	0.021^b^	0.620
	F–P	0.505	0.854	0.104	0.585	1.098^a^	1.016^a^	0.431	2.374	0.232	0.800
	H–M	0.029	0.665	0.155	0.495	0.290	0.110	0.682	0.576	0.307	0.368
	H–P	0.053	0.390	0.102	0.484	0.552	0.066	0.136	0.126^b^	0.096	0.223
	M–P	0.024^b^	0.274^b^	0.053	0.011^b^	0.262	0.176	0.546	0.450	0.212	0.223

*A–F* avian–fish, *A–H* avian–human, *A–M* avian–non-primate mammals, *A–P* avian–non-human primate, *F–H* fish–human, *F–M* fish–non-primate mammals, *F–P* fish–non-human primate, *H–M* human–non-primate mammals, *H–P* human–non-human primate, *M–P* non-primate mammals–non-human primate; ^a^largest RSCU distances among the host pairs for the corresponding codon; ^b^smallest RSCU distances among the host pairs for the corresponding codon

### COA results for RSCU values

**Fig. 10:**
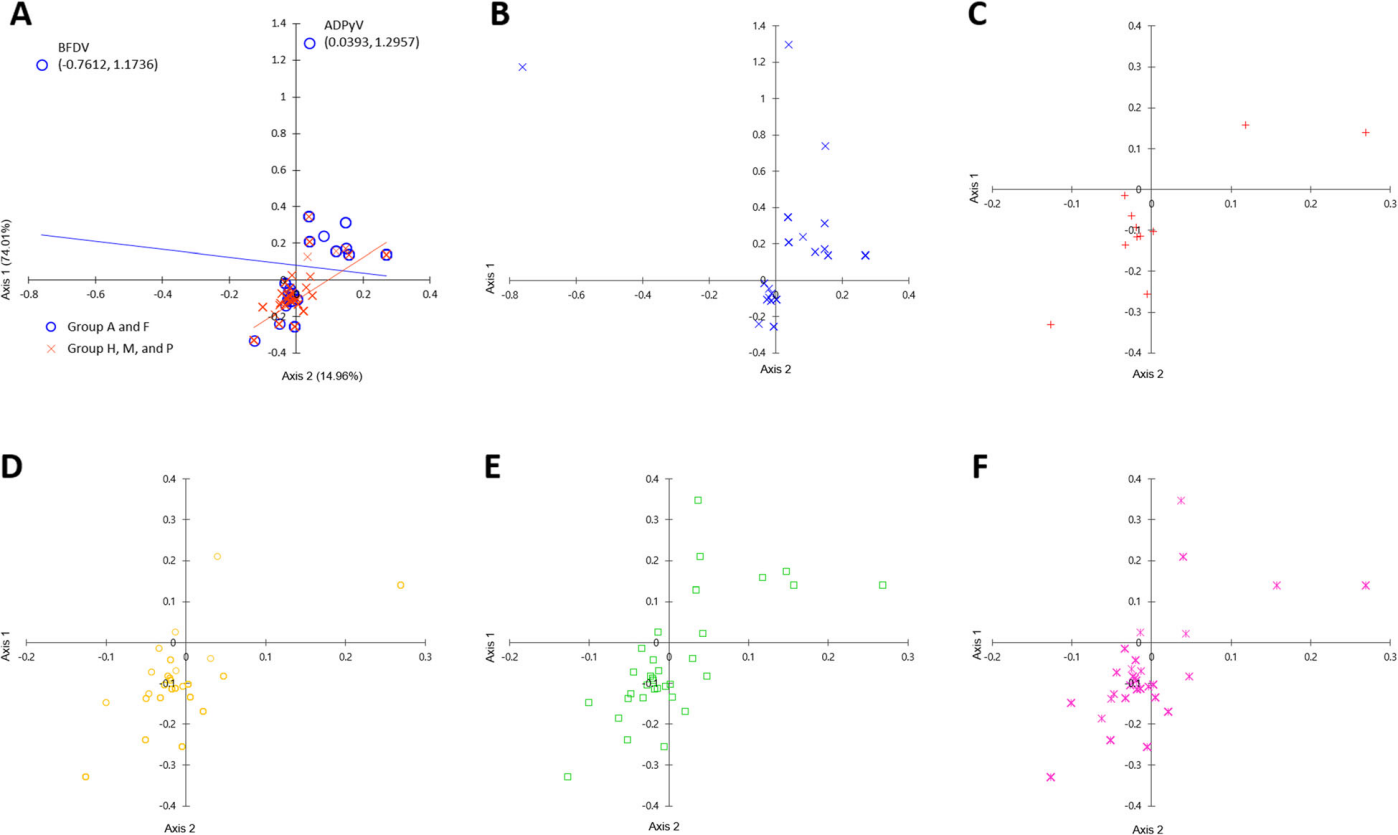
Correspondence analysis results for the RSCU values of strongly preferred codons in 86 PyVs (COA-RSCU). The COA results for over-represented codons (RSCU > 1.6) for five groups are shown in scatter plots **b**-**f** for groups A, F, H, M, and P, respectively. The plot dot distribution patterns of groups A and F vs. groups H, M, and P were compared (**a**). Overall, the plotted dots show high similarity in terms of distribution patterns in all groups, with a scattered range (− 0.2 to + 0.3, − 0.4 to + 0.4). Specifically, two dots plotted over the range were identified as *LT-Ag* genes for BFDV and ADPyV, and thus they can be seen to vary in terms of codon usage patterns. They are all avian polyomaviruses belonging to group A, and host organisms are wild birds and *Pygoscelis adeliae* (Adélie penguin) (**a**)

We carried out COA using the RSCU value to identify trends associated with differences in codon preference among the gene sequences used in this study. In the COA-RSCUs generated in this study, axis 1 (y) and axis 2 (x) accounted for 74.01 and 14.96% of the total mutations, respectively. Figure 10 shows the COA results for over-represented codons, with RSCU values greater than or equal to 1.6, calculated from 86 *LT-Ag* gene CDS. Scatter plots B–F show high similarity in terms of the distribution patterns of the plot dots in the range (− 0.2 to + 0.3, − 0.4 to ~ + 0.4) in all groups. Specifically, two dots plotted outside the corresponding range were identified as *LT-Ag* genes of BFDV and Adélie penguin PyV (ADPyV). Thus, they were presumed to indicate mutations in codon usage patterns. These are all avian PyVs belonging to Group A, and host species are wild birds and *Pygoscelis adeliae* (Adélie penguin), respectively (Fig. 10). The distances between the genes in the plots shown in Fig. 10 reflects the dissimilarity in the RSCU with respect to axis 1 and axis 2. These results explain a significant portion (74.01%) of the variation in codon usage in 86 *LT-Ag* genes, so natural selection may have played a very important role.

### Selection pressure

The dN/dS ratio was used to estimate the natural selection pressure acting on the *LT-Ag* gene. The average dN/dS values for the DnaJ and helicase domains in the overall population and in each Group (Groups A, H, M, and P for DnaJ; Groups A, F, H, M, and P for helicase) were less than 1, showing that these two functional regions experience negative selection pressure (Table 5). Similarly, negative selection pressure was estimated for LT-Ag sequence pairs within Groups A, F, M, and P, ranging from 0.282 to 0.684, while the values within the overall population and Group H exceeded 1, which suggests that human PyVs have evolved by positive selection.

## Discussion

In this study, we compared the nucleotide sequences encoding all PyV-encoded LT-Ag that have been classified so far and their major domains. Of the various virus species used for analysis, avian PyVs differed significantly from mammalian PyVs in terms of nucleotide composition, ENC value, and codon usage patterns. Avian PyVs are known to cause acute and chronic diseases in various bird species (Table 3). In particular, PyV disease [19–22], which is caused by BFDV and FPyV (finch PyV) infection, and hemorrhagic nephritis and enteritis [23], which is caused by GHPV infection, are inflammatory diseases that cause high mortality in young avians. The high virulence of these avian PyVs contrasts with mammalian PyVs, which generally cause harmless, persistent infection in natural hosts with healthy immune systems. Mammalian PyVs, such as SV40, are known to induce tumors in nonpermissive host rodents after inoculation [89], which is rarely seen in avian PyV-infected birds. In general, the avian PyV’s infectious nature, destroying numerous cells in the infected organism, is considered to cause serious diseases. The cause of significant cell damage by these viruses has not yet been elucidated. However, while avian PyV infection in chicken embryonic fibroblasts causes remarkable cell damage by induction of apoptosis, SV40 infection of Vero cells mainly causes necrosis. Thus, the induction of necrosis by avian PyVs is thought to contribute to virulence through the efficient release of virus progeny and spread across the entire organism [58]. The differences in the virulences of viruses may reflect differences in the biochemical functions of LT-Ag, which were also confirmed by the genetic and evolutionary differences observed in the *LT-Ag* gene and domains of PyVs isolated from various hosts, based on the sequence analysis performed in this study.

## Conclusions

One possible explanation for the presence or absence of specific domains or sequence motifs in the LT-Ag of various PyV species, and thus the mutations and evolutionary differences observed in these functional and structural regions, is that PyVs have evolved so that each viral protein interacts with host cell targets, and they have adapted to thrive in particular host species and cell types. They are known to interact specifically with host proteins involved in cell proliferation and gene expression regulation, have a significant association with the functional domains of LT-Ag, and vary with respect to size and composition in various virus species. Thus, even though various PyV species adopt a common survival strategy, some viral LT-Ags can target new host systems or cell types. Furthermore, the domains of LT-Ag may appear to be widely conserved, but, as indicated by the genetic and evolutionary differences observed in this study, the host function regulation mechanism of LT-Ag varies with the host species. These differences can be used to study virus–host interactions, cellular pathways, mechanisms of tumorigenesis by viral infection, and treatments for new infectious diseases. As new PyVs continue to be found in various organisms, it is necessary to conduct further studies on the mechanisms involved in host-specific toxic manifestations of PyVs, host system regulation, and cell transformation.

## Data Availability

All data and materials described in the manuscript are available.

## Abbreviations

ADPyV: Adélie penguin polyomavirus
BatPyV2c: Bat polyomavirus 2c
BatPyV4a: Bat polyomavirus 4a
BFDV: Budgerigar fledgling disease virus
BKPyV: BK polyomavirus
BPyV: Bovine polyomavirus
CAI: Codon adaptation index
CDS: Coding sequence
COA: Correspondence analysis
DPyV: Dolphin polyomavirus 1
ENC: Effective number of codons
FPyV: Finch polyomavirus
GHPV: Goose hemorrhagic polyomavirus
JCPyV: JC polyomavirus
KIPyV: KI polyomavirus
LT-Ag: Large tumor antigen
MCPyV: Merkel cell polyomavirus
ML: Maximum likelihood
MPyV: Mouse polyomavirus
PR2: Parity rule 2
PyV: Polyomavirus
RSCU: Relative synonymous codon usage
SV40: Simian virus 40
WUPyV: WU polyomavirus
